# Diversity and characterization of bacterial communities of five co‐occurring species at a hydrothermal vent on the Tonga Arc

**DOI:** 10.1002/ece3.7343

**Published:** 2021-03-10

**Authors:** Won‐Kyung Lee, S. Kim Juniper, Maëva Perez, Se‐Jong Ju, Se‐Joo Kim

**Affiliations:** ^1^ Genome Editing Research Center Korea Research Institute of Bioscience and Biotechnology Daejeon Korea; ^2^ Department of Biology School of Earth and Ocean Sciences University of Victoria Victoria BC Canada; ^3^ Département des Sciences Biologiques Université de Montréal Montreal QC Canada; ^4^ Korea Institute of Ocean Science & Technology Busan Korea

**Keywords:** bacterial community structure, Campylobacteria, chemosynthesis‐based ecosystem, Gammaproteobacteria, symbiosis, vent invertebrates

## Abstract

Host–symbiont relationships in hydrothermal vent ecosystems, supported by chemoautotrophic bacteria as primary producers, have been extensively studied. However, the process by which densely populated co‐occurring invertebrate hosts form symbiotic relationships with bacterial symbionts remains unclear. Here, we analyzed gill‐associated symbiotic bacteria (gill symbionts) of five co‐occurring hosts, three mollusks (“*Bathymodiolus*” *manusensis*, *B*. *brevior*, and *Alviniconcha strummeri*) and two crustaceans (*Rimicaris variabilis* and *Austinograea alayseae*), collected together at a single vent site in the Tonga Arc. We observed both different compositions of gill symbionts and the presence of unshared operational taxonomic units (OTUs). In addition, the total number of OTUs was greater for crustacean hosts than for mollusks. The phylogenetic relationship trees of gill symbionts suggest that γ‐proteobacterial gill symbionts have coevolved with their hosts toward reinforcement of host specificity, while campylobacterial *Sulfurovum* species found across various hosts and habitats are opportunistic associates. Our results confirm that gill symbiont communities differ among co‐occurring vent invertebrates and indicate that hosts are closely related with their gill symbiont communities. Considering the given resources available at a single site, differentiation of gill symbionts seems to be a useful strategy for obtaining nutrition and energy while avoiding competition among both hosts and gill symbionts.

## Graphical abstract

This study is the first comparison of gill symbiont communities of co‐occurring invertebrates living at a single hydrothermal vent site. We observed both different compositions of gill symbionts and the presence of unshared operational taxonomic units, as well as the representative sulfur‐oxidizing γ‐proteobacterial species which is suggested to have coevolved with their hosts toward reinforcing host specificity. Considering the given resources at a single site, differentiation of gill symbionts is a useful strategy for obtaining nutrition and energy while avoiding competition among both hosts and gill symbionts.

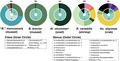

## 1 INTRODUCTION

Symbioses are ubiquitous across diverse ecosystems worldwide, with representative examples including plants with root nodule bacteria, mammals with intestinal bacteria, and aquatic animals with epibionts (Fred et al., 2002; Hooper et al., 2002; Ponnudurai, 2019). In the ocean, there are symbiotic relationships that allow organisms to share habitats and to interact with each other for benefits such as food supply and protection from predators (Beinart, 2019; Goffredi, 2010). Notably, deep‐sea hydrothermal vent ecosystems, which have light‐limited and chemical‐rich conditions, are supported by chemoautotrophic bacteria as primary producers (Corliss et al., 1979; Powell & Somero, 1986; Van Dover, 2000; Vetter, 1985). In addition, some vent invertebrates are constrained primarily by their nutritional reliance on bacterial symbionts (Cavanaugh et al., 1981; Felbeck & Childress, 1988).

Chemoautotrophic symbionts were first discovered in the vestimentiferan tubeworm *Riftia pachyptila* at hydrothermal vents along the Galapagos Rift in 1981 (Cavanaugh et al., 1981). Since then, taxonomic and biogeographic knowledge of bacterial symbionts of diverse vent organisms including mytilid mussels, provannid snails, alvinocaridid shrimps, and bythograeid crabs has steadily advanced (Duperron et al., 2006; Fujiwara et al., 2000; Goffredi, 2010; Ponnudurai, 2019; Suzuki et al., 2006; Williams, 1980; Won et al., 2003; Zbinden et al., 2008; Zhang et al., 2017). According to previous studies, bacterial symbionts densely populate specific organs and tissues of their hosts. Vestimentiferan endosymbionts occur densely in bacteriocytes within a highly vascularized internal organ, the trophosome (Jones, 1988). Meanwhile, some vent organisms, including mytilids, provannids, and alvinocaridids, contain dense aggregations of endo‐ and/or episymbionts on the gills or in branchial chambers (Distel et al., 1995; Dubilier et al., 2008; Duperron et al., 2006; Petersen et al., 2010). In terms of the interactions between hosts and symbionts, the representative symbiont of vestimentiferans, *Candidatus* Endoriftia persephone, has a broad geographic distribution as well as wide ranges of vent habitats and hosts (Di Meo et al., 2000; Perez & Juniper, 2016). In addition, some mytilid mussels show dual symbiosis, having two bacterial symbionts with different metabolic functions (Duperron et al., 2007, 2008; Jang et al., 2020). Based on these studies, the distribution, occurrence, and transmission of bacterial symbionts are assumed to be influenced by various factors, including habitat features, vent fluid composition, and the geographic distribution of their hosts (Vrijenhoek, 2010). However, previous studies have generally been conducted separately for various host species, preventing comprehensive analyses of the competitive interactions between hosts in various taxonomic groups and their symbionts.

The gill, which is one of the most extensively studied organs in relation to symbiosis, is the major organ of gas exchange and direct uptake of various organic and inorganic components from water, and its basic structure and function are similar across most aquatic animals (Riisgård, 1988; Rivera‐Ingraham et al., 2016; Wood & Soivio, 1991). The uptake rates of dissolved gases and chemical compounds are influenced by their concentrations and molecular weights, environmental conditions, and species‐specific anatomical features of the gill (Black & McCarthy, 1988; Hayton & Barron, 1990; Jørgensen, 1974; Perry & Laurent, 1993). In chemosynthetic hydrothermal vent environments, gills also function as a point of entry for highly concentrated toxic materials, such as cadmium, copper, mercury, sulfur, and methane, into the internal tissues (Cavanaugh et al., 1981; Serafim et al., 2006; Felbeck, 1981; Lee et al., 2015; Vetter, 1985). Bacterial symbionts related to gills are assumed to play key roles in supporting host metabolism and other physiological functions, such as carbon fixation, detoxification of metals, and oxidation of sulfides and methane (Cavanaugh et al., 1988; Childress et al., 1986; Jannasch, 1985; Ponsard et al., 2013; Powell & Somero, 1986; Zbinden et al., 2015).

The South‐West Pacific Area biogeographic province, our study area, covers a large area of hydrothermal vents in the southwestern Pacific Ocean. These vents are relatively young (<10 Mya) and are enriched in CO_2_, SO_2_, H_2_S, Fe, and particularly Hg compared to vent fields in other oceans (Auzende et al., 1988; Lee et al., 2015). The water masses surrounding the area are well mixed by the South Equatorial Current system (Desbruyères et al., 2006; Mitarai et al., 2016). In addition, this region is a marine biogeographic province with the highest biodiversity, as it contains diverse vent invertebrates belonging to various phyla, including mollusks, crustaceans, annelids, echinoderms, and cnidarians, which are abundant at vent sites with active hydrothermal chimneys (Bachraty et al., 2009; German et al., 2011; Thaler & Amon, 2019).

**FIGURE 1:**
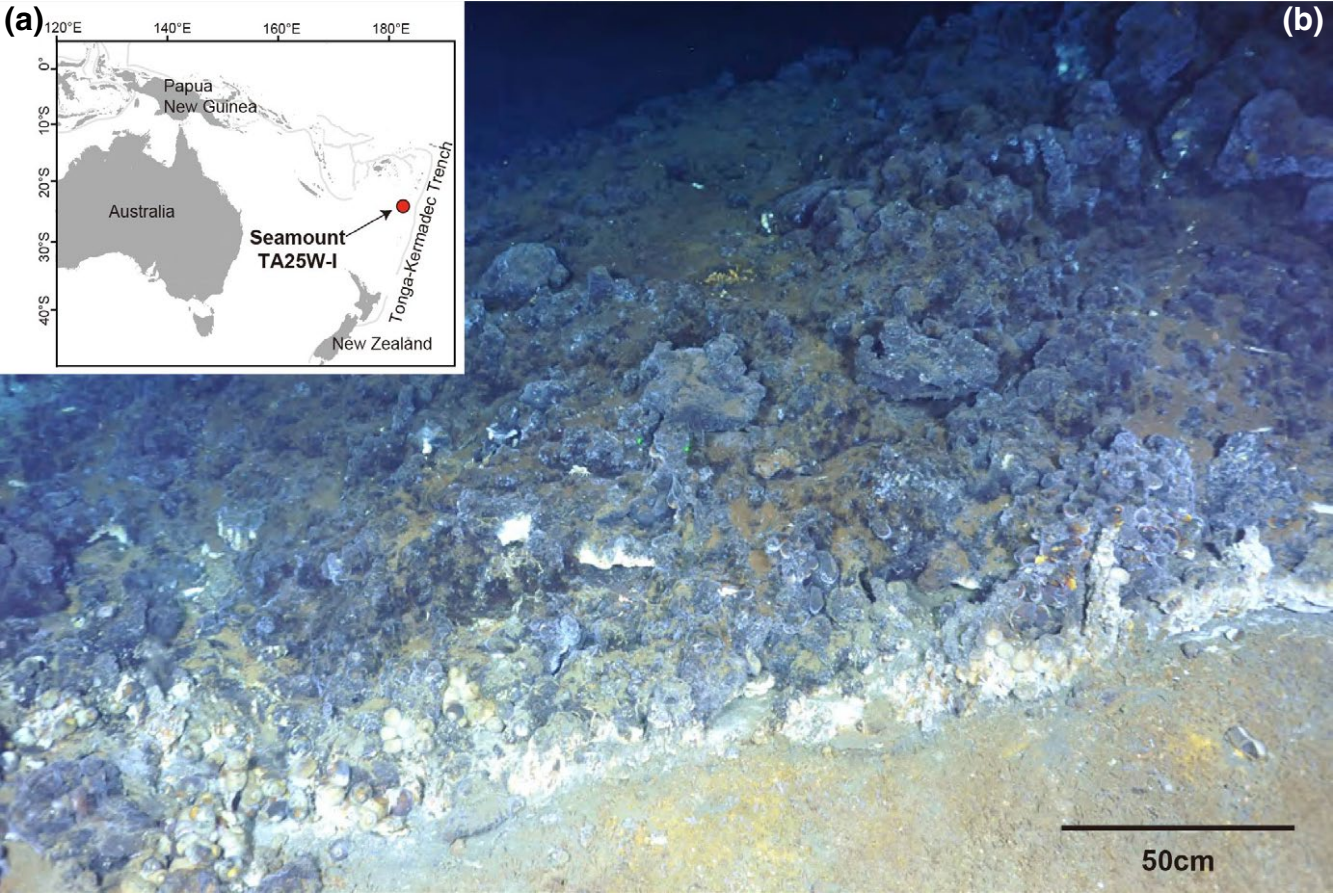
(a) Map and (b) photograph of the sample collection site in Tonga Arc. Red circle indicates the location of vent site TA25W‐I at Volcano 18S. Video clip of the collection site recorded by full HD camera mounted on ROV *ROPOS* is attached as Supplemental information

At the vent of our study site (Figure 1), five dominant co‐occurring invertebrate species, “*Bathymodiolus*” *manusensis*, *B*. *brevior*, *Alviniconcha strummeri*, *Rimicaris variabilis*, and *Austinograea alayseae*, were found under identical environmental conditions (Video S1 and Figure S1). The mechanisms by which these co‐occurring species share and partition the given resources at a single vent site remain unknown. In this study, we investigated their coexistence strategies based on gill‐associated symbiotic bacteria (gill symbionts). First, we obtained 16S rDNA libraries of gill symbionts from these five sympatric invertebrates and characterized their community compositions. To clarify the codependent relationship between gill symbionts and their hosts, we constructed phylogenetic relationship trees of the dominant gill symbionts and discussed their relationships along with the taxonomic relationships among hosts.

## 2 MATERIALS AND METHODS

### 2.1 Sample collection

Three species of mollusk (“*B*.” *manusensis*, *B*. *brevior*, *Al*. *strummeri*) and two crustaceans (*R*. *variabilis*, *Au*. *alayseae*) were collected from a single vent site TA25W‐I (referred as TA25D in Lee et al., 2019) in Volcano 18S (Massoth et al., 2007) of the Tonga Arc (24.35°S; 176.57°W; depth 1,067 m) using a scoop and suction sampler on the remotely operated vehicle (ROV) *ROPOS* during a cruise on R/V *Sonne* in February 2012 (Figure 1). Once onboard, some invertebrate specimens were immediately preserved in a −80°C deep freezer for genetic analyses.

Site TA25W‐I is part of the Tongan Exclusive Economic Zone (EEZ). Permission to conduct scientific research activity and collect biological samples in this area was granted by the Minister for Lands and Natural Resources, Kingdom of Tonga, to KIOST Minerals Limited, Korea Institute of Ocean Science and Technology.

### 2.2 DNA extraction, library preparation and pyrosequencing

We dissected gill tissues from two frozen individuals per invertebrate host species. To remove any contaminants from ships, laboratories, or humans, the dissected gill tissues were rinsed with 70% ethanol once, and then washed five times with 1× phosphate‐buffered saline (PBS). After it had been washed, genomic DNA was extracted using the FastDNA SPIN Kit for Soil (MP Biomedicals) following the manufacturer's instructions.

To prepare the metabarcoding libraries, first, the V1–V3 region of the bacterial 16S rRNA gene was amplified using the fusion primer set B16S‐*F* (5′‐CCTATCCCCTGTGTGCCTTGGCAGTC‐TCAG‐AC‐GAGTTTGATCMTGGCTCAG‐3′; underlined sequence indicates the priming site) and B16S‐R (5′‐ CCATCTCATCCCTGCGTGTCTCCGAC‐TCAG‐X‐AC‐WTTACCGCGGCTGCTGG‐3′; “X” indicates a barcode uniquely designed for each sample and the underlined sequence indicates the priming site; Table S1). PCR was conducted in 30‐µl volumes containing 1 µl genomic DNA, 4 µl dNTP mixture (2.5 mM each), 1 µl each primer (10 pmol), 3 µl 10× Ex Taq Buffer (Mg^2+^ plus), 1.25 U of Takara Ex Taq DNA Polymerase (Takara Biotechnology Co.), and 19.5 µl distilled water. The thermal cycling program was as follows: 94°C for 5 min; followed by 30 (for “*B*.” *manusensis*, *B*. *brevior*, *Al*. *strummeri*, and *Au*. *alayseae*) or 35 (for *R*. *variabilis*) cycles of 94°C for 30 s, 50°C for 30 s, and 72°C for 30 s; and finally 72°C for 7 min followed by a 20°C hold. All experiments were performed in triplicate. Next, the concentrations of the amplified products were measured using a Nanodrop 1000 Spectrophotometer (Thermo Fisher Scientific) and 300 ng of each amplified product was transferred to a single 1.5 ml microcentrifuge tube. The mixed amplified products were purified and concentrated using QIAquick Gel Extraction Kit (Qiagen, Hilden, Germany) following the manufacturer's instructions. Finally, the samples passed quality‐control testing for pyrosequencing based on a final concentration of 83 ng/µl and volume of 31 µl.

### 2.3 Data pre‐processing and OTU identification

All pyrosequencing results were subjected to Good's coverage estimation to determine the sequencing depth using CLcommunity software version 3.46. Subsequently, data pre‐processing of raw reads was conducted following the methods of Jeon et al. (2013). First, low‐quality reads (average Q score < 25 or read length < 300 bp) were discarded and the specific bacterial reads for each host sample were sorted using their unique barcodes. The barcode, linker, and PCR primer sequences were trimmed from both ends of the reads using pairwise sequence alignment and the hmm‐search program in the HMMER 3.0 package (Eddy, 2011), and chimeric sequences were removed using UCHIME (Edgar et al., 2011). Based on the clusters of trimmed sequences, which allowed no more than two mismatched bases, representative reads were selected for correcting homopolymer errors (Jeon et al., 2013). The selected representative reads were defined as OTUs and were classified using the EzTaxon‐e database. Then, taxonomic ranks were defined based on similarity values (*x*), as follows: *x* ≥ 97% for species; 97% > *x* ≥ 94.5% for genus; 94.5% > *x* ≥ 86.5% for family; 86.5% > *x* ≥ 82% for order; 82% > *x* ≥ 78.5% for class; and 78.5% > *x* ≥ 75% for phylum (Tindall et al., 2010).

### 2.4 Characterization of gill symbiont communities

Species‐level OTUs were used for subsequent analyses. Species richness and diversity were estimated with the Chao1 and Shannon indices using the Cluster Database at High Identity with Tolerance (CD‐HIT) method in CLcommunity ver 3.46 (ChunLab Inc.).

To clarify the taxonomic relationships among the dominant gill symbionts of sympatric hosts, species‐level OTUs accounting for more than 1% of each gill symbiont community were selected. These OTUs and bacterial 16S rDNA sequences (420–493 bp) associated with chemosynthetic environments were retrieved from GenBank and aligned using the Geneious Alignment method implemented in Geneious Prime v2020.0.4 (Biomatters) and further corrections were made through visual inspection. Then a neighbor‐joining tree was constructed using MEGA X (Kumar et al., 2018) with the *p*‐distance model and bootstrap resampling (1,000 replicates).

## 3 RESULTS

### 3.1 Diversity of gill symbionts from co‐occurring invertebrate hosts

A range of 4023–7170 reads of the V1–V3 region on the bacterial 16S rRNA gene were obtained from the gill tissues of three mollusks (“*B*.” *manusensis*, *B*. *brevior*, *Al*. *strummeri*) and two crustaceans (*R*. *variabilis*, *Au*. *alayseae*), with Good's coverage values >97%, which indicates sufficient sequencing depth to cover the microbial communities (Table 1; individual variations shown in Figure S2 and Table S2). The 436 total operational taxonomic units (OTUs) of gill symbionts from Tongan invertebrates included 21 bacterial phyla. The newly obtained sequences were deposited in the Sequence Read Archive (SRA) of GenBank under BioProject PRJNA637194 with following BioSample accession numbers: SAMN15098003–SAMN15098012.

**TABLE 1:** Diversity of gill symbiont communities from five co‐occurring invertebrates living at a hydrothermal vent site of the Tonga Arc based on the V1–V3 region of bacterial 16S rDNA

Host	No. of reads (mean)	No. of total OTUs^a^	No. of OTUs > 1%^b^ (% of total reads)	Chao1	Shannon
Mollusca					
“*B*.” *manusensis*	4,835	48	4 (98.5)	67.7	1.25
*B*. *brevior*	5,408	38	1 (99.3)	73.2	0.34
*Al*. *strummeri*	7,170	56	2 (97.9)	74.6	1.23
Crustacea					
*R*. *variabilis*	4,023	152	8 (88.8)	221.4	2.61
*Au*. *alayseae*	6,498	326	12 (80.5)	485.1	3.17

^a^ Threshold for species‐level OTUs was 97% similarity.

^b^ Species‐level OTUs accounting for more than 1% of reads in each gill symbiont community.

Crustacean hosts had more OTUs than mollusk hosts (Table 1). In particular, the blind crab *Au*. *alayseae* was associated with a large number of OTUs (more than 300 OTUs) relative to other species. However, in all hosts, a small number of specific OTUs accounted for more than 80% of total reads, while most OTUs had abundances of <1%. In *B*. *brevior*, a single OTU, BBG_OTU1, represented 99.3% of the total reads (Table A1).

**FIGURE 2:**
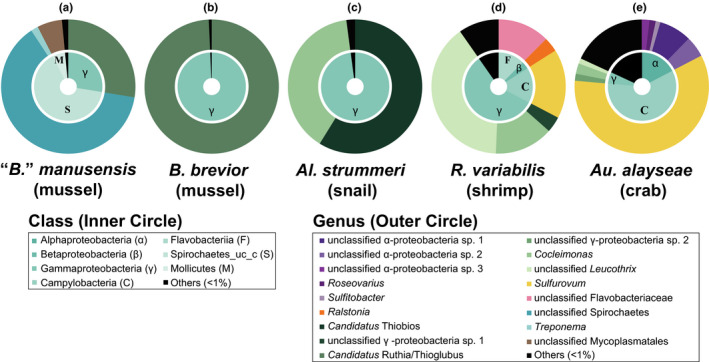
Composition of gill symbiont communities from five co‐occurring invertebrates living at a hydrothermal vent site of the Tonga Arc at the level of bacterial class and genus. Symbiont communities of the hosts (a) “*Bathymodiolus*” *manusensis*, (b) *B*. *brevior*, (c) *Alviniconcha strummeri*, (d) *Rimicaris variabilis*, and (e) *Austinograea alayseae*. Different colors represent different taxa. OTUs accounting for <1% of the community are labeled as “Others” and presented in black

The phylum Proteobacteria was the only gill symbiont taxon detected in all hosts. More specifically, γ‐proteobacterial OTUs were present in all hosts and were the only symbionts detected at levels above 1% in *B*. *brevior* and *Al*. *strummeri*, while α‐ and β‐proteobacterial OTUs were identified only in *Au*. *alayseae* and *R*. *variabilis*, respectively (Figure 2). Aside from Proteobacteria, the phylum Epsilonbacteraeota was abundant only in crustaceans (15.46% for *R. variabilis* and 57.8% for *Au alayseae*). The phyla Spirochaetes (63.2% for BMS_OTU1 and 1.76% for BMS_OTU2) and Tenericutes (6.1% for BMM_OTU1) were abundant in “*B*.” *manusensis*, while Bacteroidetes (12.2% for RVF_OTU1) was dominant in *R. variabilis*. Although previous studies have reported phenotypic characterization of these phyla (Brown, 2010; Paster & Dewhirst, 2000; Stokke et al., 2015), we were unable to enhance the discussion of these four OTUs because they showed phylogenetic uncertainties on trees based on 16S rDNA partial sequences (data not shown).

### 3.2 Relationship between gill symbionts and hosts

**FIGURE 3:**
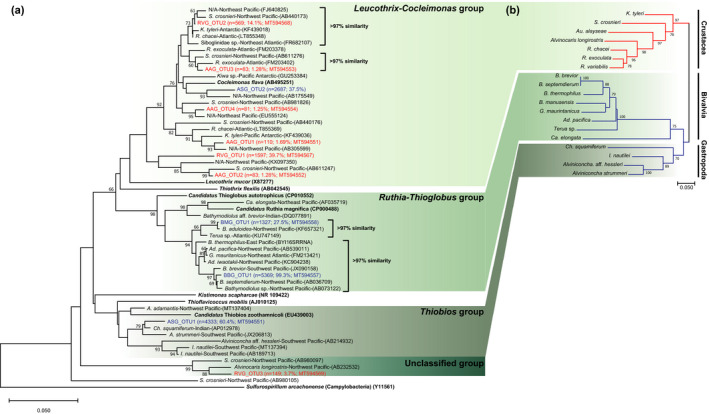
Neighbor‐joining trees based on (a) the 16S rDNA of the γ‐proteobacteria and (b) the cytochrome c oxidase subunit 1 gene of crustacean and mollusk hosts from chemosynthetic environments. Red and blue letters indicate gill symbiont OTUs from crustacean and mollusk hosts, respectively, identified in this study. OTU names are shown in Table A1. Sequences of isolated bacterial species are shown in bold. Sequences retrieved from GenBank are presented with their host, collection ocean, and GenBank accession no. Bootstrap values >60% are given above the nodes

Overall, in mollusks, the gill symbiont community was very simple, consisting of 1–4 major OTUs (Table A1). Notably, in *B*. *brevior* and *Al*. *strummeri*, the communities were mainly composed of the γ‐proteobacterial OTUs, BBG_OTU1 (99.3%), ASG_OTU1 (60.4%), and ASG_OTU2 (37.5%). On the other hand, the two sympatric *Bathymodiolus* species showed different compositions and relative abundances of gill symbionts as well as unshared OTUs. Nevertheless, their γ‐proteobacterial OTUs, BMG_OTU1 and BBG_OTU1, formed a monophyletic clade, the *Ruthia‐Thioglobus* group, and showed 96.7% similarity (Figure 3). In addition, the main OTUs from *Al*. *strummeri*, ASG_OTU1 and ASG_OTU2, clustered into different groups, the *Thiobios* and *Leucothrix*‐*Cocleimonas* groups, respectively. The former group is associated with vent snails from the Pacific Ocean (Nakagawa et al., 2014), while the latter was closely related to γ‐proteobacterial OTUs from crustacean hosts (Apremont et al., 2018; Yoshida‐Takashima et al., 2012).

**FIGURE 4:**
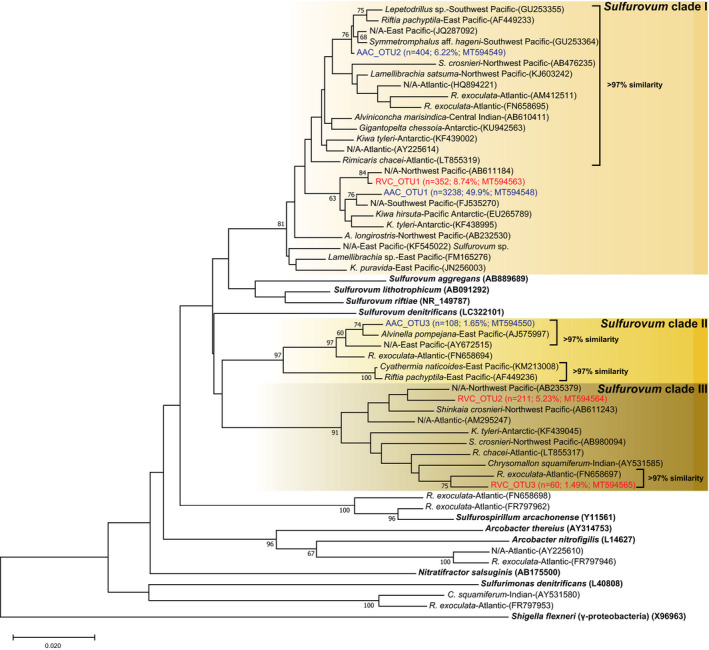
Neighbor‐joining tree based on the 16S rDNA of the Campylobacteria related with chemosynthetic environments. Red and blue letters indicate gill symbiont OTUs from *Rimicaris variabilis* and *Austinograea alayseae*, respectively, identified in this study. OTU names are shown in Table A1. Sequences of isolated bacterial species are shown in bold. Sequences retrieved from GenBank are presented with their host, collection ocean, and GenBank accession no. Bootstrap values >60% are given above the nodes

In the two crustacean hosts investigated, the complexity of the gill symbiont community exceeded that of the mollusks (Figure 2; Table A1). Nevertheless, their communities were composed of OTUs from two bacterial groups, including γ‐proteobacterial and campylobacterial OTUs (57.5% and 16.9%) in *R*. *variabilis* and α‐proteobacterial and campylobacterial OTUs (17.3% and 59.1%) in *Au*. *alayseae*. Among them, campylobacterial OTUs were found only in crustacean hosts, which contained three OTUs each. These OTUs were closely related to *Sulfurovum* species and divided into three clades, *Sulfurovum* clades I–III (Figure 4), while the γ‐proteobacterial OTUs of the crustaceans were divided into two groups, the *Leucothrix‐Cocleimonas* and unclassified groups, which contain diverse γ‐proteobacteria found in vent crustaceans or environmental samples, with the exception of ASG_OTU2.

## 4 DISCUSSION

### 4.1 Diversity of sulfur oxidizers in the gills of Tongan invertebrates

Based on metabolite uptake experiments, it has been proposed that Tongan hydrothermal vent ecosystems are supported by sulfur‐oxidizing bacteria as primary producers and inorganic sulfur compounds as their main energy source (Dubilier et al., 1998; Henry et al., 2008; Suzuki et al., 2006). In this study, the gill symbiont communities of Tongan invertebrates were mainly composed of γ‐proteobacterial sulfur oxidizers, including *Cocleimonas*, *Leucothrix*, and *Candidatus* Ruthia/Thioglobus (Figure 3; Table A1). Furthermore, in crustaceans, campylobacterial *Sulfurovum* species, which are also sulfur‐oxidizing bacteria, were dominant.

In hydrothermal vent ecosystems, γ‐proteobacteria are the most commonly observed sulfur‐oxidizing symbionts of invertebrate hosts (Apremont et al., 2018; Forget & Kim Juniper, 2013; Goffredi, 2010; Spiridonova et al., 2006). We observed γ‐proteobacterial OTUs in all five co‐occurring invertebrates from the Tonga Arc. Interestingly, none of the hosts shared species‐level OTUs that accounted for more than 1% of total reads for their symbiont community (Table A1). Based on the phylogenetic relationship tree, we assumed that the speciation of Tongan γ‐proteobacterial gill symbionts is closely related to the speciation of their hosts (Figure 3). In other words, the *Leucothrix*‐*Cocleimonas* group originated from a common ancestor that formed a symbiotic relationship with the common ancestor of brachyuran and anomuran crabs and caridean shrimps, while the ancestors of the *Ruthia*‐*Thioglobus* and *Thiobios* groups formed associations with the ancestors of mytilid‐vesicomyid bivalves and vent gastropods, respectively. These results suggest that Tongan gill symbionts are more closely affiliated with hosts than with environments. Thus, Tongan γ‐proteobacterial gill symbionts have coevolved with their hosts, and their symbiotic relationships may have been reinforced by host–symbiont interactions (e.g., bacterial chemotaxis, suppression of host immune responses, out‐competition, and cospeciation; Beinart, 2019; Charleston & Perkins, 2006; West et al., 2006) rather than through accidental acquisition due to environment factors.

The campylobacterial *Sulfurovum* is known as a representative sulfur‐oxidizing epibiont of chemosynthetic ecosystems. It grows chemolithoautotrophically using sulfur or thiosulfate as an electron donor and oxygen or nitrate as an electron acceptor (Inagaki et al., 2004). Although only four *Sulfurovum* species have been described to date, many 16S rDNA sequences closely related to *Sulfurovum* have been detected in marine sulfidic environments worldwide (Giovannelli et al., 2013; Huber et al., 2007; Inagaki et al., 2004; Mino et al., 2014). In this study, AAC_OTU2 of *Sulfurovum* clade I showed greater than 97% similarity with 14 other 16S rDNA sequences detected from diverse hosts in various regions in the global ocean (Figure 4). This result indirectly indicates that campylobacterial species with these sequences are distributed globally and have weak host preference. Moreover, a similar symbiotic relationship was revealed for members of *Sulfurovum* clades II and III, as shown in Figure 4. Based on these results, we further consider studies at the genome level to understand low variations within 16S rDNA sequences among *Sulfurovum* strains from different oceans and hosts.

### 4.2 Higher diversity of bacterial communities in crustaceans

In all Tongan invertebrates, more than 90% of bacterial OTUs in gills had abundances <1% (Figure 2). If bacterial communities of gills are mainly affected by their external environments, the level of rare OTUs should be similar in all hosts. However, the bacterial community diversities, along with the number and read ratio of rare OTUs, were extremely elevated in crustacean hosts (Table 1, Figure 2). Based on previous studies with dominant OTUs, differentiation of bacterial communities seems to be affected by symbiont forms, endosymbiont for mollusks versus episymbiont for crustaceans (Apremont et al., 2018; Duperron et al., 2007). Although this is one of the important factors for understanding bacterial communities, it is not sufficient to explain rare OTUs in this study.

In terms of animal behavior, grooming activities performed by crustaceans would have positive effect on bacterial fouling (Gebruk et al., 2000; Thurber et al., 2011). For example, according to an interesting behavioral study of the squat lobster, *Kiwa puravida*, its cheliped‐waving increases in close proximity to seeps discharging methane‐rich fluids, which is assumed to be a strategy to ensure a supply of chemical resources for the episymbionts covering its cheliped setae (Thurber et al., 2011). Generally, the necessity and usefulness of symbioses are approached from the perspective of hosts, rather than that of symbionts. Probably, from the bacterial symbionts’ viewpoint, mobile organisms may be considered better hosts than sessile ones (Micheli et al., 2002; Van Dover & Fry, 1989; Van Dover et al., 1988).

### 4.3 Competitive interaction between sympatric bathymodiolin mussels

Sympatric organisms occupying the same ecological niche generally have strategies to avoid competition for food resources and habitat (Baumart et al., 2015; Friedlaender et al., 2015). In chemosynthetic ecosystems, two bathymodiolin mussel species are occasionally found at the same sites, and such species show different gill symbiont compositions (Table 2). Interestingly, in all three cases presented in Table 2, one of the two sympatric hosts had a single symbiont metabolic type, thiotrophic in vents and methanotrophic in seeps, while the other had dual symbiont types, either methanotrophic–thiotrophic or carboxydotrophic–thiotrophic (Duperron et al., 2007; Jang et al., 2020). Generally, bathymodiolin mussels depend on their gill symbionts for nutrition (Duperron, 2010). Considering given resources available at a single site, differentiation of the gill symbionts to utilize different chemosynthetic metabolisms seems to be a brilliant strategy for sympatric bathymodiolins to peacefully obtain nutrition and energy.

**TABLE 2:** Comparison of symbiont types among sympatric bathymodiolin mussels

Ocean	Region (site, habitat)	Host Species	Symbionts^a^	Reference
West Pacific	Tonga Arc (TA25W‐I, vent)	*Bathymodiolus brevior*	T	This study
		“*Bathymodiolus*” *manusensis*	C‐T	
Indian	Central Indian Ridge (Onnuri vent field, vent)	*Bathymodiolus marisindicus*	T	Jang et al. (2020)
		*Gigantidas vrijenhoeki*	M‐T	
Atlantic	Gulf of Mexico (Alaminos Canyon, seep)	*Bathymodiolus brooksi*	M‐T	Duperron et al. (2007)
		*Gigantidas childressi*	M	

^a^ Chemosynthetic types of gill symbionts: C, carboxydotroph; M, methananotroph; T, thiotroph.

### 4.4 Minor gill symbionts of Tongan invertebrates

Most studies of bacterial community structures have focused on the predominant species in particular environments and hosts. Recently, however, a few studies have discussed the importance of rare bacteria in various natural communities, such as those in human organs, polluted soils and water, and biogas plants (Ainsworth et al., 2015; Sachdeva et al., 2019). Similarly, in chemosynthetic environments, the main research targets are thiotrophic and methanotrophic bacteria, but little is known about other rare bacteria. In this study, we observed a minor proportion of two bacterial groups, the β‐proteobacteria (3.53% for RVB_OTU1) in *R*. *variabilis* and α‐proteobacteria (7.76% for AAA_OTU1, 5.03% for AAA_OTU2) in *Au*. *alayseae* (Table A1). Although the functions of these gill symbionts remain unclear, we can assume that they cohabitate with their hosts and/or other bacteria to obtain nutrients and act as regulators of physiological processes (Dubilier et al., 2008; Duperron, 2010).

This study is the first comparison of gill symbiont communities of co‐occurring invertebrates living at a single vent site of the Tonga Arc. The results indicate that hosts are closely related with their gill symbiont communities. Thus, each host species has certain lifestyle traits, that is, it may be either sessile or mobile, filter‐feeding or predatory, and competitive or cooperative, leading to the formation of a specific symbiotic relationship between the host and symbiont. Eventually, such host–symbiont specificity would potentially reduce competition, thus promoting the coexistence of densely populated co‐occurring hosts.

Previous studies have focused on certain tissue types of specific taxa. Therefore, the process by which symbiotic relationships are formed between hosts and symbionts and the strategies used to avoid competition among host species in chemosynthetic ecosystems remains unclear. To improve our understanding of host–symbiont coevolutionary processes, further research should be conducted, including adding more host taxa found in deep‐sea environments worldwide and expansion of the targeted host organs and tissues. Furthermore, to elucidate the functions of symbionts as regulators of host physiological processes, application of genomics, as well as community structures and functions of uncultured microorganisms based on metagenomics and metatranscriptomics, should be investigated.

## CONFLICT OF INTEREST

None declared.

## AUTHOR CONTRIBUTIONS

**Won‐Kyung Lee:** Conceptualization (supporting); Data curation (equal); Formal analysis (equal); Visualization (lead); Writing‐original draft (equal). **S. Kim Juniper:** Writing‐review & editing (equal). **Maëva Perez:** Writing‐review & editing (equal). **Se‐Jong Ju:** Funding acquisition (lead); Writing‐review & editing (supporting). **Se‐Joo Kim:** Conceptualization (lead); Formal analysis (equal); Supervision (lead); Writing‐original draft (equal).

## Supporting information

Supplementary Material

Video S1

## Data Availability

Sequences from this study have been deposited in the National Center for Biotechnology Information (NCBI) Short Read Archive (SRA) under the BioProject Accession Number PRJNA637194 with following BioSample accession numbers: SAMN15098003–SAMN15098012.

## APPENDIX 1

**TABLE A1:** Taxonomic affiliations of major gill‐symbionts from five co‐occurring invertebrates living at a hydrothermal vent site of the Tonga Arc

Host	Taxonomic rank	OTU name^a^ (accession no.)	No. of reads (% of total reads)	Top Blast Hit			
				Accession no. (similarity %)	Host	Ocean	Region (habitat)
“*B*.” *manusensis*	**γ‐proteobacteria**						
	*Candidatus* Ruthia/Thioglobus	BMG_OTU1 (MT594558)	1,327 (27.5)	KF657321.1 (99.6)	*Bathymodiolus aduloides*	West Pacific	Okinawa Trough (vent)
	**Mollicutes**						
	Unclassified Mycoplasmatales sp.	BMM_OTU1 (MT594559)	295 (6.10)	FJ456769.1 (86.7)	*Notothenia coriiceps*	Southern	Antarctic (cold water)
	**Spirochaetes_uc**						
	Unclassified Spirochaetes sp.	BMS_OTU1 (MT594560)	3,055 (63.2)	AB645303.1 (97.0)	N/A	West Pacific	Shimokita Peninsula (sediment)
	*Treponema*	BMS_OTU2 (MT594561)	85 (1.76)	AB645303.1 (96.8)	N/A	West Pacific	Shimokita Peninsula (sediment)
*B. brevior*	**γ‐proteobacteria**						
	*Candidatus* Ruthia/Thioglobus	BBG_OTU1 (MT594557)	5,369 (99.3)	AP013042 (99.6)	*Bathymodiolus septemdierum*	West Pacific	Myojin Knoll (vent)
*Al. strummeri*	**γ‐proteobacteria**						
	*Candidatus* Thiobios	ASG_OTU1 (MT594555)	4,333 (60.4)	AP012978 (98.7)	*Chrysomallon squamiferum*	Indian	Central Indian Ridge (vent)
	*Cocleimonas*	ASG_OTU2 (MT594556)	2,687 (37.5)	AB175549 (96.5)	N/A	West Pacific	Okinawa Trough (vent)
*R. variabilis*	**β‐proteobacteria**						
	*Ralstonia*	RVB_OTU1 (MT594562)	142 (3.53)	MK934373 (99.6)	N/A	N/A	Cultured
	**γ‐proteobacteria**						
	Unclassified *Leucothrix* sp. 1	RVG_OTU1 (MT594567)	1,597 (39.7)	KX097350 (93.0)	N/A	West Pacific	Okinawa Trough (vent)
	*Cocleimonas*	RVG_OTU2 (MT594568)	569 (14.1)	LT855348 (99.2)	*Rimicaris chacei*	Atlantic	Mid‐Atlantic Ridge (vent )
	Unclassified γ ‐proteobacteria sp. 1	RVG_OTU3 (MT594569)	149 (3.70)	AB232532 (96.8)	*Alvinocaris longirostris*	West Pacific	Okinawa Trough (vent)
	**Campylobacteria**						
	*Sulfurovum*	RVC_OTU1 (MT594563)	352 (8.74)	AB611184 (99.1)	N/A	West Pacific	Okinawa Trough (vent)
		RVC_OTU2 (MT594564)	211 (5.23)	AM295247 (97.6)	N/A	Atlantic	Mid‐Atlantic Ridge (vent )
		RVC_OTU3 (MT594565)	60 (1.49)	FN658697 (97.8)	*Rimicaris exoculata*	Atlantic	Mid‐Atlantic Ridge (vent )
	**Flavobacteriia**						
	Unclassified Flavobacteriaceae sp.	RVF_OTU1 (MT594566)	492 (12.2)	FN662579 (97.9)	*Rimicaris exoculata*	Atlantic	Mid‐Atlantic Ridge (vent )
*Au. alayseae*	**α‐proteobacteria**						
	Unclassified α‐proteobacteria sp. 1	AAA_OTU1 (MT594543)	504 (7.76)	AF449223 (99.3)	*Riftia pachyptila*	East Pacific	East Pacific Rise (vent)
	Unclassified α‐proteobacteria sp. 2	AAA_OTU2 (MT594544)	327 (5.03)	DQ856534 (94.0)	*Eriocheir sinensis*	West Pacific	China (river estuary)
	*Roseovarius*	AAA_OTU3 (MT594545)	98 (1.51)	AB121095 (98.7)	N/A	West Pacific	Japan sea (cold seep)
	*Sulfitobacter*	AAA_OTU4 (MT594546)	74 (1.14)	NR_165714 (100)	N/A	West Pacific	Northwest Pacific (deep water)
	Unclassified α‐proteobacteria sp. 3	AAA_OTU5 (MT594547)	120 (1.85)	NR_118566 (99.8)	N/A	West Pacific	South sea of Korea (sediment)
	**γ‐proteobacteria**						
	Unclassified γ‐proteobacteria sp. 2	AAG_OTU1 (MT594551)	110 (1.69)	AB305599 (97.6)	N/A	West Pacific	Okinawa Trough (vent)
	Unclassified *Leucothrix* sp. 2	AAG_OTU2 (MT594552)	83 (1.28)	AB611247.1 (97.6)	*Shinkaia crosnieri*	West Pacific	Okinawa Trough (vent)
	*Cocleimonas*	AAG_OTU3 (MT594553)	83 (1.28)	AB611276 (99.0)	*Shinkaia crosnieri*	West Pacific	Okinawa Trough (vent)
		AAG_OTU4 (MT594554)	81 (1.25)	EU555124 (98.57)	N/A	East Pacific	Juan de Fuca Ridge (vent)
	**Campylobacteria**						
	*Sulfurovum*	AAC_OTU1 (MT594548)	3,238 (49.9)	FJ535270 (97.9)	N/A	West Pacific	Kermadec (vent)
		AAC_OTU2 (MT594549)	404 (6.22)	JQ287092 (99.6)	N/A	East Pacific	East Pacific Rise (vent)
		AAC_OTU3 (MT594550)	108 (1.65)	AY672515 (98.3)	N/A	East Pacific	East Pacific Rise (vent)

^a^ The OTU name consists of four parts: the first letter of the generic name of the host + the first letter of specific epithet of the host + the first letter of the class name of the gill‐symbiont + a number assigned in descending order of read count at the level of bacterial class.
