# Supplementary material for: Diversity and characterization of bacterial communities of five co‐occurring species at a hydrothermal vent on the Tonga Arc
Source: Ecol Evol. 2021 Mar 10;11(9):4481–93. doi: 10.1002/ece3.7343 (PMC8093707; doi:10.1002/ece3.7343)
Supplement: Supplementary file 1 — Supplementary Material [file ECE3-11-4481-s001.docx]

**Supplemental Information for:**

**Diversity and characterization of bacterial communities of five co-occurring species at a hydrothermal vent on the Tonga Arc**

Won-Kyung Lee^a^, S. Kim Juniper^b^, Maëva Perez^c^, Se-Jong Ju^d#^, Se-Joo Kim^a#^

**Table of Contents:**

| **Figure S1** | Page 1 |
| --- | --- |
| **Figure S2** | Page 2 |
| **Table S1** | Page 3 |
| **Table S2** | Page 4 |
| **Table S3** | Page 5 |
| **Video clip** | Separate file |

**
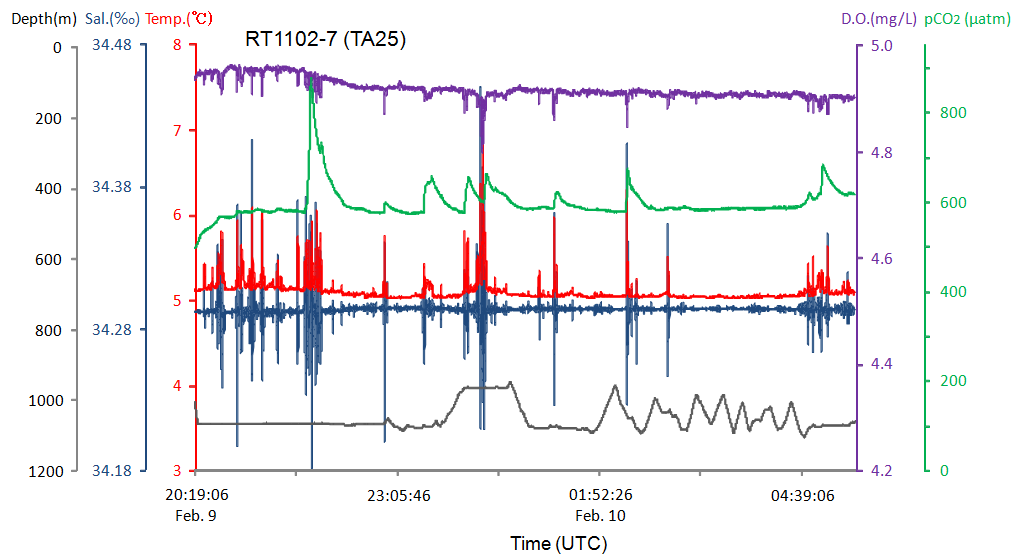
**

**Figure S1. Depth, salinity, temperature, dissolved oxygen concentration, and carbondioxide concentration of the sampling site TA25 measured by sensors mounted on ROV *ROPOS* during the expedition. Yellow box presents specific sampling point for biological specimens.**


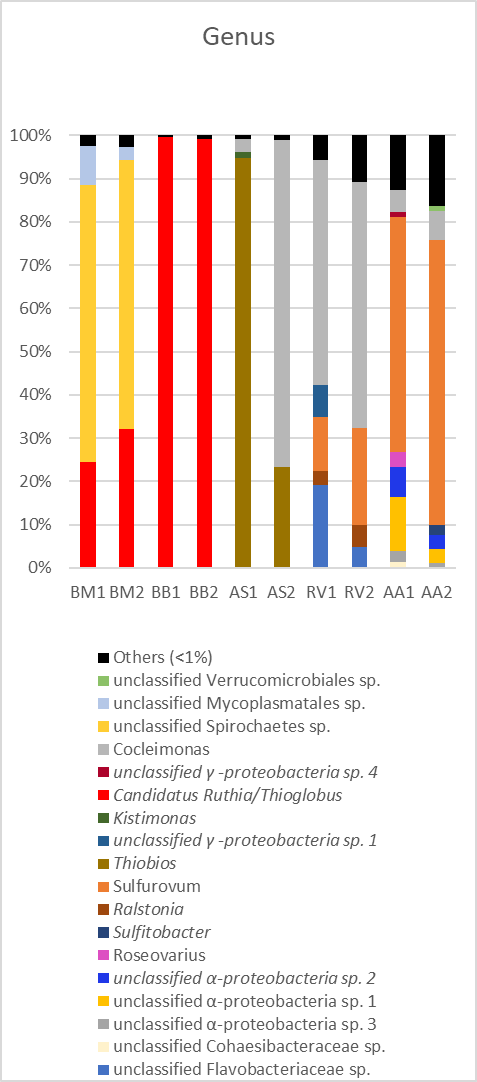

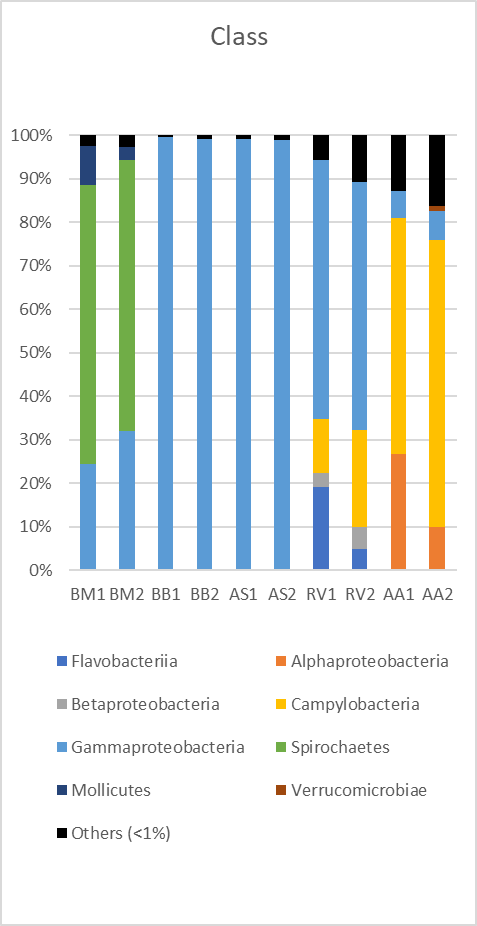


**A**

**B**

**Figure S2. Composition of gill-symbiont communities from two individuals per invertebrate host species from the Tonga Arc at the level of bacterial class (A) and genus (B). Sample symbol consists of three parts: the first letter of the generic name of the host + the first letter of specific epithet of the host + the individual number of the host.**

| **Table S1. List of primers with specific barcodes** | | | | |
| --- | --- | --- | --- | --- |
|  | Name | Adapter-Key | Barcode ('X') | Linker-Primer |
| Forward primer | B16S-F | CCTATCCCCTGTGTGCCTTGGCAGTC-TCAG | N/A | AC-GAGTTTGATCMTGGCTCAG |
| Reverse primers | BM1_Gill | CCATCTCATCCCTGCGTGTCTCCGAC-TCAG | ATCGTGTG | AC-WTTACCGCGGCTGCTGG |
|  | BM2_Gill |  | CTACACAG |  |
|  | BB1_Gill |  | TAGCTACG |  |
|  | BB2_Gill |  | TCGAGTAG |  |
|  | AS1_Gill |  | CGTGTACTG |  |
|  | AS2_Gill |  | CTGTCTACG |  |
|  | RV1_Gill |  | ATCGTCTGTG |  |
|  | RV2_Gill |  | ATCGTAGCAG |  |
|  | AA1_Gill |  | TAGATAGTGCG |  |
|  | AA2_Gill |  | ACGTCTCTACG |  |

| **Table S2. Diversity of gill-symbiont community from two individuals per invertebrate host species from the Tonga Arc based on the V1–V3 region of the bacterial 16S rDNA.** | | | | | | |
| --- | --- | --- | --- | --- | --- | --- |
| **Species** | **Sample** | **Valid reads** | **No. of OTUs** | **Chao1** | **Shannon** | **Good's Coverage (%)** |
| “*B*.” *manusensis* | BM1 | 4994 | 45 | 60.6 | 1.22 | 99.7 |
|  | BM2 | 4675 | 51 | 74.8 | 1.28 | 99.6 |
| *B. brevior* | BB1 | 4927 | 28 | 35.2 | 0.29 | 99.8 |
|  | BB2 | 5889 | 48 | 111.3 | 0.39 | 99.6 |
| *Al. strummeri* | AS1 | 7494 | 55 | 75.0 | 1.28 | 99.8 |
|  | AS2 | 6846 | 57 | 74.1 | 1.18 | 99.8 |
| *R. variabilis* | RV1 | 3858 | 124 | 169.0 | 2.59 | 98.8 |
|  | RV2 | 4187 | 180 | 273.8 | 2.63 | 98.1 |
| *Au. alayseae* | AA1 | 6409 | 274 | 391.4 | 3.24 | 98.3 |
|  | AA2 | 6586 | 377 | 578.9 | 3.11 | 97.5 |

**Table S3. dbRDAs for evaluating host related-variables which significantly affect barcterial community composition**

| **Variables evaluated** | **Proportion of variance (%)** | **Df** | **SumsOfSqs** | ***F*-value** | ***p*-value** |
| --- | --- | --- | --- | --- | --- |
| Species | 92 | 4 | 6.06 | 14.34 | 0.001 |
| Mobility† | 67 | 2 | 4.43 | 7.19 | 0.003 |
| Taxon group†† | 47 | 1 | 3.07 | 6.97 | 0.007 |
| Replicates | 2 | 4 | 0.16 | 0.11 | 0.971 |

^†^sessile, grazing, or swimming/walking

^††^mollusks or crustaceans
